# Validating Temporal Eye Tracking Metrics as Orthogonal Biomarkers for Aggressive Traits: A Mixed-Effects Analysis

**DOI:** 10.3390/jemr19030044

**Published:** 2026-04-28

**Authors:** Omar Alvarado-Cando, Oscar Casanova-Carvajal, José-Javier Serrano-Olmedo

**Affiliations:** 1 Psychology Brain and Innovation in Neuroscience Group, Facultad de Informática y Ciencias de la Computación, Universidad Católica de Cuenca, Cuenca 010107, Ecuador; 2 Centro de Tecnología Biomédica (CTB), Campus de Montegancedo, Universidad Politécnica de Madrid, 28223 Madrid, Spain; oscar.casanova@ctb.upm.es; 3 Departamento de Ingeniería Eléctrica, Electrónica, Automática y Física Aplicada, Escuela Técnica Superior de Ingeniería y Diseño Industrial ETSIDI, Universidad Politécnica de Madrid, 28040 Madrid, Spain; 4 Centro de Investigación Biomédica en Red para Bioingeniería, Biomateriales y Nanomedicina, Instituto de Salud Carlos III, 28040 Madrid, Spain

**Keywords:** eye tracking, biomarker, aggressive profile, emotion, GLM

## Abstract

Atypical visual attention to aversive or threatening stimuli is a clinically relevant feature of aggressive behavior. However, the developmental dissociation between sustained visual allocation and early orienting remains unclear. This study examined the temporal dynamics of visual attentional biases in a sample of 119 children and adolescents (51 males, 68 females), clinically and behaviorally categorized into aggressive and non-aggressive cohorts. Using a free-viewing paradigm with standardized emotional stimulus pairs selected from the International Affective Picture System (IAPS), eye-tracking analysis focused on first-fixation direction and dwell time. Inferential analyses were conducted using Linear Mixed-Effect Models (LMM) and Generalized Linear Mixed-Effects Models (GLMM). The linear model revealed a significant main effect of behavioral condition: individuals with aggressive traits, regardless of their stage of development, showed greater sustained visual allocation toward negative stimuli. In contrast, the GLMM for first-fixation direction identified a significant age-by-condition interaction, indicating that early orienting differences were more clearly expressed in the aggressive adolescent cohort. These findings suggest that sustained visual preference for negative content may represent a relatively stable correlate of aggressive traits, whereas early orienting differences may vary across developmental stages. Together, these two temporal eye-tracking measures may provide complementary information for future computational approaches to aggression screening. In conclusion, these two temporal oculomotor dimensions may provide a useful feature space for future machine-learning pipelines and may serve as complementary candidate markers for comparing computational predictions against clinically established ground truth in aggression screening research.

## 1. Introduction

Aggressive behavior in youth represents a major public health problem, deeply rooted in maladaptive patterns of social information processing (SIP). According to the SIP model, aggressive individuals tend to interpret ambiguous social signals as hostile, a phenomenon known as Hostile Attribution Bias [1,2]. Traditionally, the clinical and forensic assessment of this trait has relied heavily on standardized self-report questionnaires and observational scales. While instruments such as the Buss-Perry Aggression Questionnaire [3], the Brøset Violence Checklist (BVC) [4], and the Dynamic Appraisal of Situational Aggression (DASA) [5] have demonstrated strong reliability and predictive validity for imminent violence [6], their reliance on subjective appraisal limits their scalability and mechanistic insight into the cognitive underpinnings of aggression.

Recent neurocognitive evidence suggests that aggressive behavior is not merely a high-level cognitive error but is based on early-stage visual attentional deficits [7,8,9]. Specifically, the visual system of aggressive individuals can be tuned to preferentially detect and maintain attention on threatening stimuli, reinforcing a cycle of hostile cognition and reactive behavior [10,11,12]. Eye-tracking studies have demonstrated that individuals with higher hostility traits exhibit systematic differences in visual attention, including selective attention to negative or violent stimuli and altered orienting to socially relevant cues [13,14,15,16]. However, visual attention is not a unitary process. It comprises two distinct temporal components governed by different neural networks: (i) Initial Orienting (bottom-up), a rapid, automatic capture mechanism driven by the salience of the stimulus and mediated by the amygdala and striatal circuits; and (ii) Attentional Maintenance (top-down), a controlled, sustained engagement regulated by the prefrontal cortex (PFC) to disengage from emotional distractors [17,18,19]. In the context of aggression, distinguishing between these two mechanisms is critical.

Despite the theoretical clarity of these two attentional mechanisms, the current literature presents conflicting results on eye-tracking classification, mainly due to small sample sizes and the aggregation of data across wide age ranges. We argue that this developmental aggregation masks critical physiological differences. Adolescence is characterized by a neurodevelopmental mismatch in which limbic structures associated with emotional reactivity mature earlier than the prefrontal regions responsible for cognitive control [20,21]. Consequently, the visual processing of social information is not static; attentional biomarkers of aggression in childhood can differ fundamentally from those that consolidate during adolescence. Thus, the developmental stage must be modeled as a critical interacting variable.

Beyond developmental confounders, previous research has often suffered from methodological heterogeneity. Methodological best practices explicitly recommend defining theoretical Areas of Interest (AOIs) and relying on explicit fixation metrics rather than large exploratory batteries [22,23,24]. From a biomedical engineering perspective, overcoming these limitations is crucial to identifying objective physiological features that can serve as a reliable input for automated classification systems. Although functional neuroimaging (fMRI) and wearable biosensors demonstrate moderate to strong predictive validity for aggression [25,26], they are often cost-prohibitive or anatomically restrictive. Conversely, temporal eye-tracking metrics, specifically fixation onset and dwell time, offer a non-invasive, high-resolution avenue to quantify these biases. Although current eye-tracking measures have shown significant but modest associations with aggressive tendencies as standalone metrics [22], integrating them as orthogonal dimensions could greatly improve computational classification

This study aims to disentangle the temporal dynamics of visual attention in aggressive youth by applying a mixed-effects modeling approach to a free-viewing paradigm with standardized emotional stimuli (IAPS). We tested the hypothesis that deficits in top-down regulation (attentional maintenance) are stable trait-like features of aggression, whereas bottom-up hypervigilance (initial orienting) is a developmental pattern that consolidates during adolescence. By validating these orthogonal oculomotor dimensions against clinical Ground Truth, this research seeks to establish a robust feature space for future AI-assisted diagnostic tools.

## 2. Materials and Methods

This study adopted an observational analytic case–control design conducted within the Ecuadorian educational system. The methodology framework was designed to systematically characterize oculomotor differences between pre-stratified behavioral groups (Aggressive vs. Non-Aggressive), without manipulation of aggression levels. The design enables examination of the association between the aggression profile and the attentional biomarkers while minimizing intervention-related bias.

### 2.1. Participant

A total of 119 students participated in this study, recruited from primary and secondary schools and organized into two balanced age cohorts. All participants underwent a rigorous multi-informant clinical assessment to determine aggression levels, conducted by licensed mental health professionals (clinical psychologists and neuropsychologists). The diagnostic protocol was based on methodological triangulation integrating: (i) standardized psychometric scores obtained from validated aggression scales, (ii) a structured interview with parents or guardians to assess behavioral history, and (iii) clinical corroboration provided by the Department of Psychology of the educational institution.

The child cohort comprises 60 individuals (23 males, 37 females) aged 6–10 years (M=7.8, SD=1.2), including 30 participants classified as Aggressive and 30 classified as Non-aggressive. The adolescent cohort comprises 59 individuals (28 males, 31 females) aged 13–16 years (M=14.6, SD=1.7), including 29 participants classified as Aggressive and 30 classified as Non-aggressive.

Aggression levels were evaluated using two age-appropriate, validated Spanish instruments. The use of separate scales is theoretically necessary because the phenotypic manifestation of aggression evolves across neurodevelopmental stages. For the child cohort, we used the Cuestionario de Agresividad Física y Verbal (AFV) [27], a validated Spanish translation of the Physical and Verbal Aggression Scale [28], which demonstrated strong internal consistency (Cronbach’s α=0.83). For the adolescent cohort, we utilized the Cuestionario de Agresividad Premeditada e Impulsividad en Adolescentes (CAPI-A) [29], a natively Spanish instrument formally validated for Latin American youth, which also yielded high reliability (Cronbach’s α=0.83). Based on the multi-source evaluation framework, participants were classified into two behavioral groups: “Aggressive” and “Non-aggressive”, according to the clinical criteria.

Written informed consent was obtained from parents or legal guardians, and assent was provided by the participants. The study adhered to the Declaration of Helsinki and was approved by the Institutional Review Board (UCCUE-CEISH-2024-045).

### 2.2. Stimuli

The visual stimuli consisted of 32 standardized photographs selected from the International Affective Picture System (IAPS) [30,31]. Because IAPS images are distributed under restricted-use agreements, the original stimuli are not reproduced in this manuscript. To facilitate reproducibility, the IAPS catalog numbers, normative ratings, and trial pairings used in the experiment will be provided in the Supplementary Materials.

Stimulus selection was based on the normative affective ratings reported in the IAPS manual. Positive images were sampled from stimuli with normative valence scores ranging from 6 to 9, whereas negative images were sampled from stimuli with valence scores ranging from 1 to 4. Neutral images (valence = 5) were excluded. Furthermore, because the sample included children and adolescents, additional exclusion criteria were applied to remove images containing explicit sexual content or extreme graphic gore; when necessary, excluded images were replaced by the next eligible image within the same valence range.

All selected images were standardized to identical pixel dimensions and presented at a uniform visual angle. In addition, the 16 stimulus pairs were computationally evaluated to minimize systematic differences in low-level visual properties between the positive and negative images within each pair. Paired comparisons confirmed no significant between-category differences in algorithmic saliency based on the Itti-Koch model [32] (p=0.957), visual entropy (p=0.500), and mean luminance (p=0.075).

### 2.3. Apparatus

Visual stimuli were displayed on a 27-inch LCD monitor (1920 × 1080 pixels; 60 Hz) (LG Electronics Inc., Pyeongtaek-Si, Republic of Korea). The presentation of the stimuli and the experiment were programmed in Gazepoint Analysis (v6.9.0) and Gazepoint Control (v6.9.0). Eye movements were recorded at 150 Hz using a desk-mounted video-oculography system, the GP3 HD (Gazepoint Research, Vancouver, BC, Canada). This device operates using corneal reflection and dark pupil detection techniques to estimate the gaze vector relative to the screen coordinates, using a high-resolution image sensor and a dedicated processing unit. The setting was carefully controlled by conducting the task in a light-proof room.

### 2.4. Procedure

**Figure 1:**
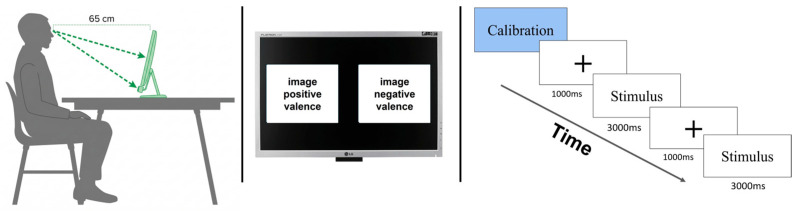
Experimental setup and trial structure. (**left**) Participant position and viewing distance relative to the monitor and eye tracker. (**middle**) Schematic representation of the stimulus display, showing the simultaneous presentation of two paired images, one on the left and one on the right side of the screen. (**right**) Temporal structure of trials: a central fixation cross presented for 1000 ms, followed by the simultaneous presentation of the paired images for 3000 ms.

Participants were seated at a fixed viewing distance of 65 cm from the display, in accordance with the manufacturer’s technical recommendations. The eye-tracking sensor was positioned at the bottom of the display monitor, remaining unobtrusive throughout the session. To minimize motor interference and attentional bias, participants did not have access to a keyboard or mouse, and manual responses were not required, Figure 1 left.

Each session began with a 9-point eye tracker calibration to ensure optimal spatial accuracy. After calibration, participants received standardized verbal instructions explaining the visual task. Specifically, they were informed that pairs of images would appear on the screen and that they were free to observe them naturally, without any specific viewing constraints or response requirements.

The experiment followed a free-viewing paradigm composed of 16 trials. Each trial (Figure 1 right) began with a central fixation cross presented for 1000 ms, followed immediately by the simultaneous presentation of two paired IAPS images for 3000 ms, one displayed on the left side of the screen and the other on the right side. In each pair, one image belonged to the negative-valence category and the other to the positive-valence category (Figure 1 middle). To reduce laterality effects and anticipatory scanning strategies, the left-right position of the positive image was randomized across trials.

### 2.5. Data Preprocessing

Raw gaze data were processed using a structured preprocessing pipeline implemented in Python (v3.13) to ensure signal integrity and derive the eye-movement variables used in the inferential analyses. AOIs were predefined based on stimulus valence and spatial boundaries. Each trial contained two AOIs, one corresponding to the positive image and the other to the negative image, resulting in a total of 32 AOIs across the 16 trials.

Initial data cleaning focused on transient losses of the eye-tracking signal, particularly blinks. To improve the validity of dwell time estimates, blink-related artifacts were identified using the raw hardware variables Blink ID and Blink Duration. In the custom preprocessing pipeline, the duration of any blink occurring while gaze was assigned to an AOI was subtracted from that AOI’s cumulative dwell time. This correction ensured that dwell time estimates reflected active visual engagement rather than signal interruption.

For latency-related preprocessing, trials in which an AOI was never fixated were coded as missing values (NaN) for that AOI’s Time to First Fixation (TTFF). No interpolation procedure was applied to these missing values, because the absence of a fixation in a free-viewing trial was interpreted as a true absence of visual allocation to that AOI rather than as a value requiring reconstruction.

A stringent trial validity criterion was then applied. Trials were excluded when the cumulative dwell time was 0 ms for both AOIs, indicating that neither image received active visual inspection during the 3000 ms presentation window. Only valid trials were retained for subsequent inferential analyses.

After quality control, the validated trial-level dataset was used to derive two complementary outcome variables: a continuous measure of sustained visual allocation based on dwell time and a binary measure of early orienting based on the direction of the first valid fixation.

#### 2.5.1. Attention Maintained Bias (Dwell Time)

Sustained visual allocation was quantified using dwell time within the two AOIs presented in each trial. For each participant and each trial, a trial-level Normalized Bias Index (NBI) was computed to express the relative distribution of dwell time between the positive and negative images while controlling for differences in total viewing time across trials [33,34]. Thus, the NBI was calculated within subjects at the trial level, and the resulting repeated observations were subsequently modeled using a mixed-effects framework.

The NBI was defined as:

(1) $$NBI=\frac{Dwell_{Positive}-Dwell_{Negative}}{Dwell_{positive}+Dwell_{Negative}}$$

This index ranges from −1 to +1, where negative values indicate relatively greater dwell time on the negative stimulus, positive values indicate relatively greater dwell time on the positive stimulus, and values near 0 indicate a more balanced distribution of viewing time between the two images. This transformation was used to capture relative attentional preference rather than absolute look duration.

#### 2.5.2. Initial Orientation Measure (First Fixation Direction)

Early orienting was operationalized as the direction of the first valid fixation within each trial. Although Time to First Fixation (TTFF) was extracted for each AOI during preprocessing, it was not used as the inferential outcome variable. Instead, TTFF served only to identify which AOI received the earliest valid fixation during the stimulus display. The AOI with the shorter valid TTFF was therefore classified as the first-fixated AOI.

Based on this rule, a binary trial-level variable was created for inferential analysis: 1 = positive first look, when the positive-valence AOI received the first valid fixation, and 0 = negative first look, when the negative-valence AOI received the first valid fixation. Trials in which neither AOI received any fixation were excluded from this analysis because the first-fixation direction could not be determined.

### 2.6. Statistical Analysis

Before inferential modeling, the analytical structure of the eye-tracking dataset was considered at the trial level. Because each participant contributed repeated observations across the 16 experimental trials, the resulting data had a hierarchical structure in which trials were nested within subjects, violating the independence assumption required by conventional fixed-effect parametric approaches. In addition, Shapiro–Wilk tests conducted on aggregated subject-level data indicated that both the temporal maintenance bias (W=0.94, p<0.001) and the initial orienting probabilities (W=0.95, p<0.001) significantly deviated from normality. Therefore, mixed-effects modeling was selected as the most appropriate inferential framework, as it allows the simultaneous estimation of population-level fixed effects and subject-specific random variability. This rationale is consistent with previous GLMM-based methodological approaches used for repeated observations and non-normal behavioral outcomes [35].

All statistical procedures were implemented in R (R Foundation for Statistical Computing, Vienna, Austria) using the package lme4 [36]. Attentional maintenance, operationalized as the continuous Normalized Bias Index (NBI), was analyzed using an LMM estimated with Restricted Maximum Likelihood (REML). In contrast, initial orientation, defined as a binary trial-level outcome (positive first look vs. negative first look), was analyzed using a GLMM with a binomial error distribution and a logit link function. Model fit was summarized using the Akaike Information Criterion (AIC) and the Bayesian Information Criterion (BIC), which are likelihood-based information criteria that quantify the trade-off between goodness-of-fit and model complexity; lower values indicate a more parsimonious fit when comparing models estimated on the same dataset. The use of these criteria is consistent with prior GLMM-based analyses of complex behavioral data, where AIC and BIC were employed to compare competing models and reduce the risk of overfitting [35].

In both modeling frameworks, age cohort (child vs. adolescent), behavioral condition (aggressive vs. non-aggressive), and their interaction were specified as fixed effects, while Subject ID was included as a random intercept to account for baseline between-subject heterogeneity in oculomotor behavior. To facilitate the interpretation of model coefficients and interaction terms, treatment contrast coding was applied. Accordingly, the reference level for the intercept was defined as the aggressive adolescent group.

Because standard lme4 objects do not report *p*-values for LMM fixed effects, statistical significance was estimated using the Satterthwaite approximation as implemented in the lmerTest package [37]. Finally, when significant interaction effects were detected, post hoc pairwise comparisons and Estimated Marginal Means (EMMs) were computed using the emmeans package [38], with adjustment for multiple comparisons.

## 3. Results

Prior to inferential modeling, a quality control procedure was applied to the gaze dataset. For the 119 participants who completed 16 experimental trials, the total expected number of trials was 1904. Trials were excluded when no fixation was allocated to either the positive or negative AOI during the entire 3000 ms stimulus window (resulting in a trial-level dwell time of 0 ms). This pattern typically reflects momentary track loss or off-screen viewing, which is common in pediatric eye-tracking paradigms.

Exactly 37 trials were excluded, yielding a retention rate of 98.06% (1867 valid trials). Most excluded trials were concentrated in the child cohort (36 trials), whereas only one excluded trial was observed in the adolescent cohort. Within the child cohort, excluded trials represented 3.33% of the total available trials in the aggressive group (16/480) and 4.17% in the non-aggressive group (20/480), indicating no meaningful imbalance in trial exclusion by behavioral condition. No additional minimum viewing-time threshold was imposed to exclude trials.

Global visual engagement metrics for the 1867 retained trials indicated good overall task engagement. On average, participants required 0.79 s to direct their gaze to one of the target images following the disappearance of the fixation cross. Furthermore, the mean active dwell time within the predefined AOIs was 2.064 s per trial, indicating that participants devoted approximately 68% of the 3000 ms stimulus period to active visual exploration of the paired images.

A Pearson correlation analysis showed a strong positive association between the dwell time bias and fixation-count bias (r=0.95, p<0.001). Because both measures indexed the same underlying dimension of visual allocation, the inferential analysis of attentional maintenance was performed using the continuous dwell time metric (NBI-Time) only, thereby avoiding redundant modeling and unnecessary inflation of the family-wise error rate.

### 3.1. Attentional Maintenance Bias (Dwell Time)

An LMM was fitted to the trial-level NBI values to examine the effects of age cohort and behavioral condition on sustained visual allocation. The fixed effects are summarized in Table 1. The model showed that the fixed effects accounted for a modest proportion of the variance (marginal $R^{2}=0.02$), which increased after including the random intercept for participants (conditional $R^{2}=0.10$).

**Figure 2:**
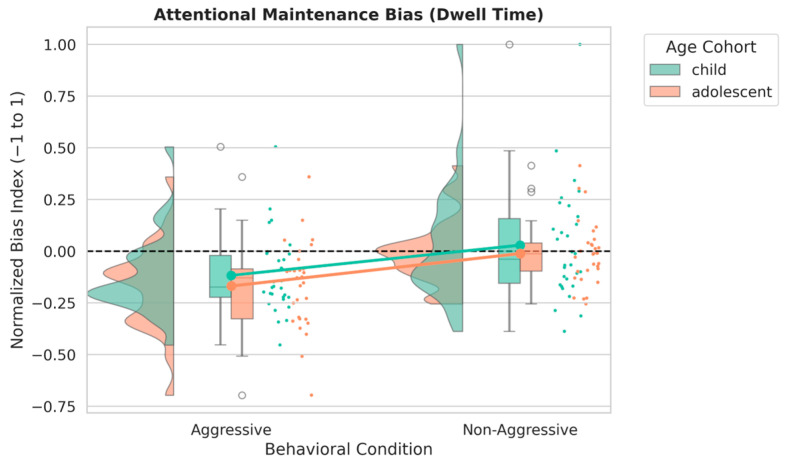
Raincloud plot illustrating the Normalized Bias Index (NBI) for dwell time as a function of behavioral condition and age cohort. More negative NBI values indicate relatively greater dwell time on the negative stimulus. The aggressive group shows a downward shift in NBI values across both developmental stages.

As detailed in Table 1, the intercept for the reference group (aggressive adolescents) was significantly below zero (β=−0.17, SE=0.04, t=−4.65, p<0.001), indicating relatively greater dwell time on the negative stimulus within this group. As shown in Figure 2, a significant main effect of behavioral condition was also observed, such that non-aggressive participants showed NBI values that were, on average, 0.16 units higher than those of aggressive participants (β=0.16, SE=0.05, t=3.08, p=0.003). This pattern indicates that participants with aggressive traits allocated relatively more sustained viewing time to negative stimuli than their non-aggressive peers.

No significant main effect of age cohort was found (β=0.05, SE=0.05, t=1.02, p=0.308), and the interaction between age cohort and behavioral condition was not statistically significant (β=−0.03, SE=0.07, t=−0.45, p=0.654). These results indicate that the association between aggressive traits and greater sustained visual allocation toward negative stimuli was similar in both children and adolescents.

### 3.2. Initial Orientation Bias (First Fixation Direction)

A GLMM with a binomial distribution and logit link function was fitted to the trial-level first-fixation direction outcome (positive first look vs. negative first look) to examine whether early orienting differed as a function of age cohort and behavioral condition. The fixed effects are summarized in Table 2.

As shown in Table 2, the intercept for the reference group (aggressive adolescents) was significantly negative (β=−0.37, SE=0.09, z=−3.88, p<0.001), indicating that, within this group, the probability of first looking at the positive stimulus was below 0.50. A significant main effect of behavioral condition was also observed (β=0.29, SE=0.13, z=2.18, p=0.029), showing that non-aggressive participants were more likely than aggressive participants to direct their first fixation toward the positive image.

The main effect of age cohort did not reach statistical significance (β=0.22, SE=0.13, z=1.65, p=0.098). However, the age cohort × behavioral condition interaction was significant (β=−0.41, SE=0.19, z=−2.20, p=0.028), indicating that the association between behavioral condition and first-fixation direction varied across developmental stages.

**Figure 3:**
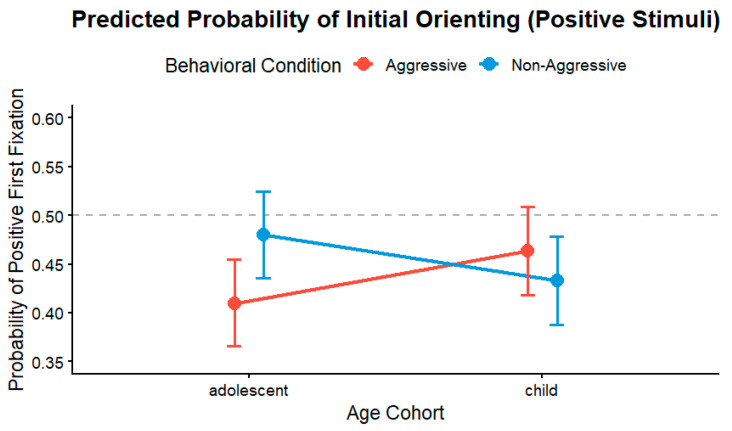
Predicted probability of first-fixation direction as a function of age cohort and behavioral condition. The *y*-axis represents the probability that the first valid fixation was directed toward the positive stimulus. The dashed line at 0.50 indicates equal probability of first orienting toward either image. Values below 0.50 indicate a relatively higher probability of first orienting toward the negative stimulus. Error bars represent 95% confidence intervals.

Estimated marginal means (EMMs) were computed on the logit scale and subsequently back-transformed into predicted probabilities to facilitate interpretation of this interaction. As illustrated in Figure 3, the interaction between age cohort and behavioral condition revealed a developmentally moderated pattern of first-fixation direction. Within the child cohort, predicted probabilities for aggressive and non-aggressive participants clustered near chance level and exhibited overlapping confidence intervals (Aggressive: 0.46, 95% CI [0.42, 0.52]; Non-Aggressive: 0.43, 95% CI [0.39, 0.48]; OR = 1.13, z = 0.94, *p* = 0.347). This pattern indicates no clear group difference in initial orienting during childhood.

However, during adolescence, the developmental trajectories diverged. Although the non-aggressive adolescent group maintained a probability close to equal orienting toward either image (0.48, 95% CI [0.44, 0.53]), aggressive adolescents showed a lower probability of first looking at the positive image (0.41, 95% CI [0.37, 0.46]). This contrast was statistically significant (OR = 0.75, z = −2.18, *p* = 0.029), indicating that adolescents with aggressive traits showed a relatively greater tendency to orient first toward the negative stimulus.

Taken together, these findings suggest that group differences in early orienting were more clearly expressed in adolescence than in childhood.

**Figure 4:**
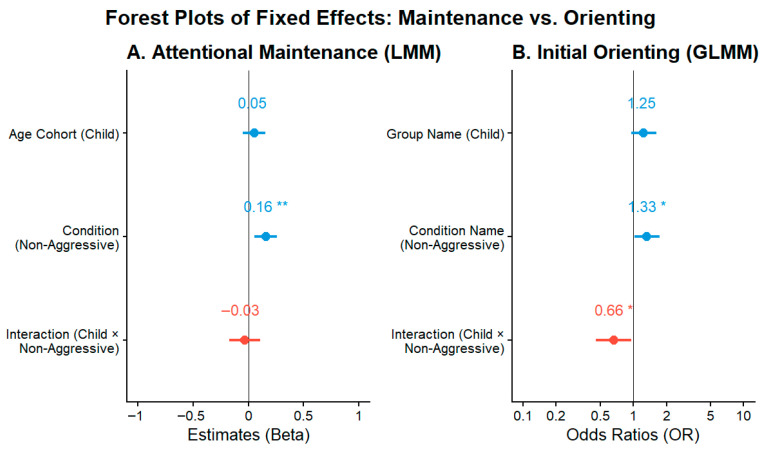
Forest plots summarize the fixed effects of the inferential models. (**A**) LMM predictors for attentional maintenance bias (beta coefficients). (**B**) GLMM predictors for first-fixation direction (odds ratios). The figure contrasts the main effect of behavioral condition on dwell time allocation with age cohort × behavioral condition observed for first-fixation direction. Asterisks indicate statistical significance (* *p* < 0.05, ** *p* < 0.01).

Figure 4 summarizes the magnitude, direction, and statistical significance of the fixed effects across both inferential models. In Panel A, behavioral condition is the only significant predictor of dwell time bias, whereas neither age cohort nor the interaction term reaches significance. In Panel B, the interaction between age cohort and behavioral condition is the key effect associated with first-fixation direction. Viewed together, these models indicate a dissociation between sustained visual allocation and initial orienting. Aggressive traits were associated with relatively greater dwell time on negative stimuli in both age cohorts, whereas differences in first-fixation direction showed developmental specificity, being more clearly expressed in adolescence than in childhood. This pattern suggests that sustained viewing preference for negative content may represent a more stable correlate of aggressive traits, whereas early orienting differences may vary across developmental stages.

## 4. Discussion

The present study examined developmental differences in two temporal components of visual attention in relation to aggressive traits: sustained visual allocation, indexed by dwell time bias, and early orienting, indexed by first-fixation direction. The results indicate a dissociation between these two components. Greater sustained allocation of gaze toward negative stimuli was observed in aggressive participants across both age cohorts, whereas differences in first-fixation direction were more clearly expressed during adolescence than during childhood. These findings support the view that temporally distinct eye-tracking measures may capture complementary aspects of socio-emotional information processing associated with aggressive traits.

The present findings are consistent with prior evidence linking aggressive behavior to biased processing of socio-emotional information. From a Social Information Processing (SIP) perspective, aggressive individuals tend to exhibit hostile attribution patterns and preferential allocation of attention to negative or threatening cues [13,39,40]. In this context, the current results suggest that aggressive traits are associated with a more sustained visual preference for negative content, while early orienting differences appear to vary as a function of developmental stage.

### 4.1. Sustained Visual Allocation Toward Negative Stimuli

The LMM showed that aggressive participants, regardless of their developmental stage, displayed more negative Normalized Bias Index (NBI) values than non-aggressive participants, indicating relatively greater dwell time on negative images. In behavioral terms, this pattern suggests a more sustained allocation of visual attention toward negative content in individuals with aggressive traits.

This finding is consistent with the prior literature linking aggression to biased processing of socio-emotional information and preferential attention to aversive or threatening cues [13,39,40]. From a neurocognitive perspective, sustained visual exploration of emotionally salient material is often discussed in relation to regulatory processes involving prefrontal and limbic systems [41]. However, because the present study employed a free-viewing paradigm without explicit task goals or competing task-relevant targets, these data should be interpreted cautiously. More specifically, the current results provide evidence of sustained visual preference for negative stimuli rather than a direct demonstration of a measured inability to disengage.

Importantly, the absence of a significant age cohort × behavioral condition interaction indicates that this pattern was present in both children and adolescents. From a developmental perspective, this suggests that greater sustained visual allocation toward negative socio-emotional information may represent a relatively stable correlate of aggressive traits across these age ranges. Repeated preferential engagement with negative content may, in turn, contribute to maladaptive patterns of interpretation and response, including hostile attribution tendencies described in the aggression literature.

### 4.2. Developmental Differences in Initial Orienting

In contrast to the dwell time findings, the GLMM for the first fixation direction indicated that early orienting patterns associated with aggressive traits were developmentally moderated. Specifically, the difference between aggressive and non-aggressive participants was more evident during adolescence, whereas children from both behavioral groups showed more similar first-fixation probabilities.

These findings should also be interpreted within the limits of the present paradigm. In a free-viewing condition, participants were allowed to look freely at either image, and no task-relevant target competed with a distractor. Therefore, the observed pattern is more appropriately described as a relative bias in early orienting toward negative stimuli rather than as definitive evidence of attentional capture or involuntary hypervigilance. This distinction is important because the present design supports inferences about preferential visual allocation, but not about failure to suppress attention to task-irrelevant material.

Within that interpretive boundary, the adolescent pattern remains theoretically relevant. The more pronounced tendency of aggressive adolescents to orient first toward the negative image may be compatible with developmental accounts proposing a mismatch between heightened socio-emotional reactivity and still-maturing regulatory systems during adolescence [42]. In this sense, the present data do not demonstrate that early orienting toward threat is newly acquired during adolescence, but they do indicate that group differences in early orienting are more clearly expressed at that developmental stage. This developmental divergence is consistent with dual-systems models suggesting that subcortical sensitivity to affective cues may outpace the maturation of top-down regulatory networks during early adolescence [18].

In addition, the computational matching of low-level visual properties across positive and negative images reduces the likelihood that these results were driven solely by systematic differences in saliency, luminance, or entropy. If the early orienting pattern were explained primarily by trivial sensory asymmetries, a more generalized bias would be expected across age and behavioral groups. Instead, the selective adolescent effect supports the interpretation that these differences were associated with stimulus valence in interaction with behavioral condition, rather than with low-level image properties alone.

### 4.3. Limitations

Although the findings are revealing, several limitations must be acknowledged. First, although the stimulus bank was standardized using 32 images from the IAPS, its static nature may not fully capture the dynamic complexity of real-world social interactions. Future studies could employ dynamic stimuli, video-based paradigms, or immersive Virtual Reality (VR) environments to increase ecological validity [43].

Second, while the cross-sectional sample size (N = 119) was sufficient for the mixed-effects models used, a larger longitudinal cohort would be necessary to determine whether the developmental differences observed in first-fixation direction reflected stable maturational change over time. The present data support developmental variation, but they do not permit causal conclusions about how these attentional patterns emerge.

Finally, the study did not include complementary physiological measures such as heart rate variability, electrodermal activity, or pupillary reactivity. Including such signals in future work could help clarify whether the observed visual biases co-occur with broader autonomic or affective response profiles.

### 4.4. Future Directions and Computational Implications

The recent literature emphasizes that predictive biosignal models achieve their highest validity (AUCs of 0.80–0.98) when personalized or capture multidimensional features [44,45]. In this context, identifying temporally distinct eye-tracking features may help advance the development of objective screening approaches in mental health. Future work could expand the current oculomotor feature space by incorporating nonlinear temporal descriptors and physiological dynamics. For example, recent studies by Calà et al. have shown the potential of Recurrence Quantification Analysis (RQA) on eye-tracking data to characterize implicit processing [46], as well as leveraging pupillary dilation dynamics to indirectly measure autonomic arousal and emotional activation during interactive tasks [47]. Integrating such measures with the dwell time and first-fixation metrics examined here may provide a richer feature architecture for future predictive models.

**Figure 5:**
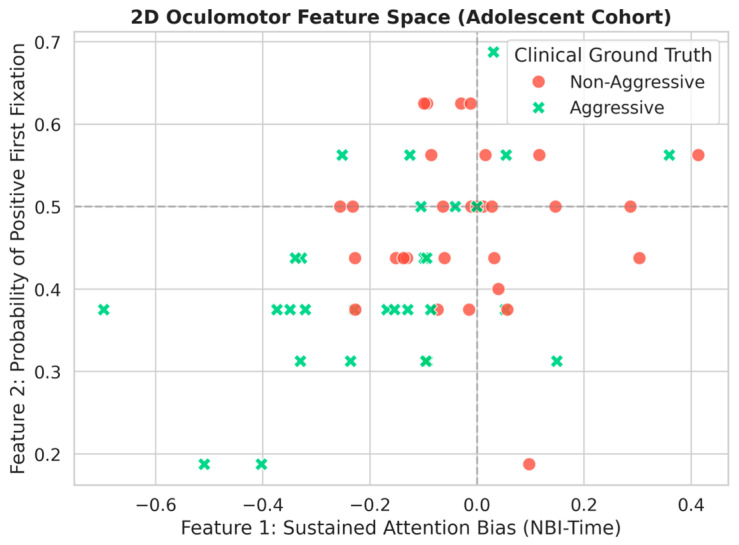
Two-dimensional oculomotor feature space for the adolescent cohort, plotting dwell time bias (*x*-axis) against initial orienting (probability of positive first fixation; *y*-axis). Data points represent individual participants, with markers indicating the clinical grouping (Aggressive vs. Non-Aggressive).

Figure 5 illustrates the adolescent cohort in a two-dimensional oculomotor feature space. By plotting dwell time bias against the probability of first looking at the positive image, a descriptive group tendency can be observed, with aggressive adolescents more frequently occupying the lower-left region of the feature space. This region is characterized by relatively greater dwell time on negative stimuli and a lower probability of initially orienting toward the positive image.

Although this visualization should be interpreted as descriptive rather than definitive, it suggests that these two temporal eye-tracking measures may provide distinct and potentially complementary information for future computational modeling. In that sense, the present findings suggest that combined fixation and dwell-based features may be useful components of future machine-learning pipelines aimed at assisting the identification of aggressive traits. Such approaches should be validated prospectively and contrasted against clinically established ground truth before any translational implementation is considered.

## 5. Conclusions

This study provides oculomotor evidence that visual attention patterns associated with aggressive traits differ across temporal components and developmental stages. Greater sustained visual allocation toward negative stimuli was observed in aggressive participants across both age cohorts, whereas differences in early orienting were more evident during adolescence. These findings support the potential value of the Normalized Bias Index and first-fixation probability as complementary eye-tracking markers for future computational models aimed at assisting aggression screening. Further prospective validation will be necessary to determine their predictive and clinical applicability.

## Figures and Tables

**Table 1:** Fixed effects for the Linear Mixed-Effects Model on attentional maintenance bias.

Predictor	Estimate (β)	SE	*t*	*p*
Intercept (Adolescent, aggressive)	−0.17	0.04	−4.65	<0.001
Behavioral Condition (Non-Aggressive)	0.16	0.05	3.08	0.003
Age Cohort (Child)	0.05	0.05	1.02	0.308
Behavioral Condition × Age (Non-Aggressive × Child)	−0.03	0.07	−0.45	0.654
Model Fit Indices				
AIC	2892.37			
BIC	2925.56			
Marginal R^2^/Conditional R^2^	0.02/0.10			

**Table 2:** Fixed effects for the generalized linear mixed-effects model on first-fixation direction.

Predictor	Estimate (β)	SE	*z*	*p*
Intercept (Adolescent, aggressive)	−0.37	0.09	−3.88	<0.001
Behavioral Condition (Non-Aggressive)	0.29	0.13	2.18	0.029
Age Cohort (Child)	0.22	0.13	1.65	0.098
Behavioral Condition × Age (Non-Aggressive × Child)	−0.41	0.19	−2.20	0.028
Model Fit Indices				
AIC	2571.29			
BIC	2598.95			
Marginal R^2^/Conditional R^2^	0.004/0.004			

## Data Availability

All the data will be free to access in this site: https://zenodo.org/records/15694220 (accessed on 23 April 2026), along with the code used in the paper.

## Supplementary Materials

The following supporting information can be downloaded at: https://www.mdpi.com/article/10.3390/jemr19030044/s1, Table S1: IAPS catalog numbers, normative ratings, and trial pairings.
