# Supplementary material for: Validating Temporal Eye Tracking Metrics as Orthogonal Biomarkers for Aggressive Traits: A Mixed-Effects Analysis
Source: J Eye Mov Res. 2026 Apr 28;19(3):44. doi: 10.3390/jemr19030044 (PMC13214924; doi:10.3390/jemr19030044)
Supplement: Supplementary file 1 [file jemr-19-00044-s001.zip › jemr-4192118-supplementary.pdf]

| Table S1: IAPS catalog numbers, normative ratings, and trial pairings. | | | |
|---|---|---|---|
| Slide No. | Valence Mean SD | Arousal Mean SD | Dominance Mean SD |
| 2110 | 3.71(1.82) | 4.53(2.25) | 4.66(2.25) |
| 4100 | 6.11(1.66) | 4.39(1.75) | 5.93(1.71) |
| 9940 | 1.62(1.20) | 7.15(2.24) | 2.45(2.22) |
| 8206 | 6.43(1.75) | 6.41(2.19) | 5.19(2.04) |
| 3213 | 3.63(1.57) | 6.89(1.55) | 4.49(2.06) |
| 9041 | 2.98(1.58) | 4.64(2.26) | 4.38(2.34) |
| 9300 | 2.26(1.76) | 6.00(2.41) | 4.12(2.57) |
| 7440 | 6.19(1.81) | 4.96(2.08) | 6.19(1.84) |
| 8510 | 7.62(1.54) | 5.44(2.58) | 6.35(2.34) |
| 9183 | 2.00(1.38) | 6.07(2.16) | 3.27(1.77) |
| 5875 | 6.16(1.61) | 3.24(2.22) | 6.12(1.97) |
| 6230 | 2.06(1.59) | 7.56(1.96) | 2.15(2.09) |
| 9156 | 6.43(1.59) | 5.79(2.30) | 6.04(2.05) |
| 5830 | 7.37(1.80) | 4.98(2.40) | 5.68(1.93) |
| 6571 | 3.80(2.16) | 5.22(2.24 | 4.26(2.27) |
| 2750 | 2.55(1.19) | 4.55(1.66) | 4.69(2.10) |
